# Nine new species of *Clada* from Madagascar (Coleoptera, Ptinidae)

**DOI:** 10.3897/zookeys.806.21916

**Published:** 2018-12-13

**Authors:** Petr Zahradník, Miloš rýzna

**Affiliations:** 1 Forestry and Game Management Research Institute, Strnady 136, CZ-150 00 Praha 5-Zbraslav, Czech Republic Forestry and Game Management Research Institute Prague Czech Republic; 2 Czech University of Life Sciences, Faculty of Forestry and Wood Sciences, Department of Forest Protection and Entomology, Kamýcká 1176, CZ-165 21 Praha 6-Suchdol, Czech Republic Czech University of Life Sciences Prague Czech Republic

**Keywords:** Afrotropical region, *
Clada
*, Coleoptera, Madagascar, new species, Ptinidae, taxonomy

## Abstract

Nine new species of the genus *Clada* (s. str.) Pascoe, 1887 (Bostrichoidea: Ptinidae: Eucradinae) are described from Madagascar: Clada (Clada) barclayi**sp. n.**, C. (C.) dimbyi**sp. n.**, C. (C.) fasciata**sp. n.**, C. (C.) lalae**sp. n.**, C. (C.) madagascarensis**sp. n.**, C. (C.) mamyi**sp. n.**, C. (C.) njakai**sp. n.**, C. (C.) obesa**sp. n.**, and C. (C.) rindrai**sp. n.** No species of this genus were previously known from Madagascar. Photographs of the dorsal habiti and drawings the male and female antennae and aedeagi of most of these species are given.

## Introduction

Madagascar is a large island (almost 600 mil. km^2^) with diverse natural conditions influenced by various geographical and climatic conditions, and also by an exceptionally rich tree flora. Most species of Ptinidae are xylophagous or fungivorous. No recent papers on the family Ptinidae from this region have been published, except the subfamily Ptininae (Bellés 1987, 1991; Philips 2005).

Many descriptions of species (almost all of them endemics in Madagascar, a few of them also occur on some neighbouring islands or in continental Africa, and a few are widely distributed or cosmopolitan) are known from older descriptions by M. Pic and some other authors. These descriptions tend to be very short, without pictures, only some of them are modern with illustrations, especially of the aedeagus. Madagascar’s fauna of Ptinidae is surely richer. This is our first contribution on this family from Madagascar.

The subfamily Eucradinae LeConte, 1861 contains two tribes, Eucradini LeConte, 1861, with the North American genus *Eucrada* LeConte, 1861 and Hedobiini Mulsant et Rey, 1868, with five genera distributed worldwide, *Anhedobia* Nakane, 1963, *Clada* Pascoe, 1887, *Hedobia* Dejean, 1821, *Neohedobia* Fisher, 1919 and *Ptinomorphus* Mulsant et Rey, 1868. Sexual dimorphism is typical of all species in the tribe Eucradini. Males have more pectinate antennae, and females less pectinate. Genera in the tribe Hedobiini have slightly serrate antennae. Only the genus *Clada* is atypical, with serrate antennae in both sexes in some species, while in others, the antennae of the male are pectinate, and those of the female serrate, and in some species antennae are pectinate in both sexes. White (1974) placed this genus in the subfamily Dryophilinae LeConte, 1861, while other authors have put it in the subfamily Eucradinae. The lateral edge of the pronotum in the subfamily Eucradinae is absent, but the subfamily Dryophilinae has the lateral edge distinct. Moreover, genera of the subfamily Dryophilinae have filiform antennae.

The genus *Clada* (Eucradinae: Hedobiini) contains two subgenera, *Taiwanoclada* Sakai, 1987 from Taiwan with only one species, and the nominal subgenus with 50 species from the Palaearctic, Oriental, and Afrotropical regions. From the sub-saharan African and southern African regions, the following species are known:

C. (C.) basilewskyi Español, 1969 Tanzania

C. (C.) costipennis Kolbe, 1897 Tanzania

C. (C.) flabellicornis Pic, 1936 Zaire

C. (C.) granulata Español, 1972 South Africa

C. (C.) humeralis Pic, 1926 Congo, Kenya, Tanzania

C. (C.) laticollis Pic, 1947 Ethiopia, Kenya

C. (C.) lineatipennis Pic, 1926 Ivory Coast

C. (C.) longicornis Pic, 1934 Kenya

C. (C.) multistriata Pic, 1952 Benin

C. (C.) rugosa Pic, 1915 Benin, Ivory Coast

C. (C.) waterhousei Pascoe, 1887 South Africa

In Madagascar, no species of the genus *Clada* were known. Overall, only 48 species and subspecies of Ptinidae are known from Madagascar, 18 from the subfamily Ptininae, one from Ernobiinae, five from Anobiinae, seven from Xyletininae, seven from Mesocoelopodinae, and ten from Dorcatominae (Español 1969a; Pic 1896, 1912a, b, 1949, 1952).

## Materials and methods

We have studied all the original descriptions of species in the subgenus Clada from Central and South Africa and also some other descriptions from neighbouring countries (including India, with some similar species) (Español 1969b, 1972; Kolbe 1897; Pascoe 1887; Pic 1915, 1926, 1934, 1936, 1947, 1952). Specimens of new species have been given a red printed label with the following text: “Holotype” or “Paratype”. On the second white printed label is the following text: “name of species. sp. n., P. Zahradník et M. Trýzna det.”.

The type materials are deposited in the following collection:

**NHMUK** Natural History Museum, London, U.K.

**MTDC** Miloš Trýzna collection, Děčín, Czech Republic

**FGMRI** Forestry and Game Management Research Institute, Jíloviště, Czech Tepublic (P. Zahradník)

**LBVC** Lukáš Blažej collection, Varnsdorf, Czech Republic

## Descriptions

### Clada (Clada) barclayi sp. n.

Taxon classificationAnimaliaColeopteraPtinidae

http://zoobank.org/3024EF07-9FDF-4F02-AC93-8AE41CCEB249

**Figures 1–9.**
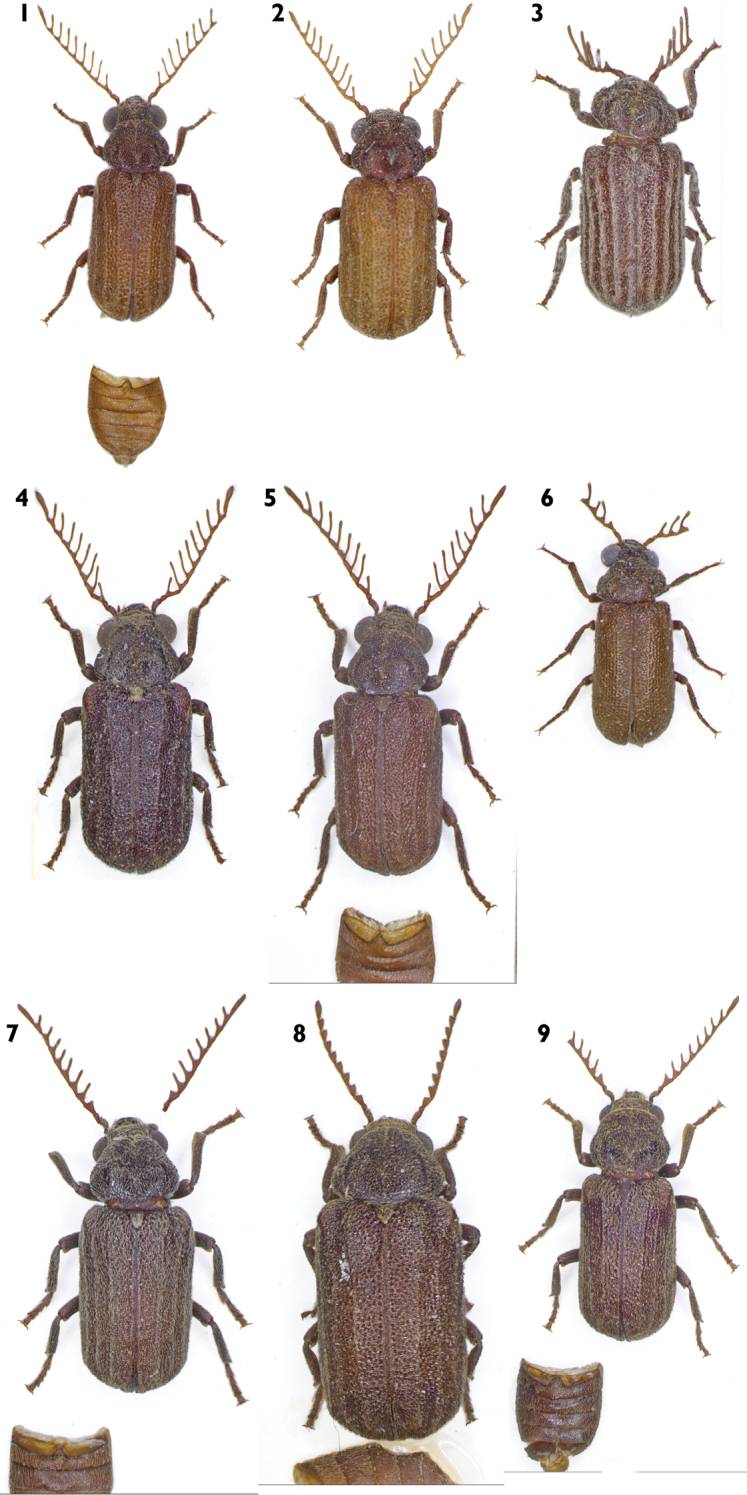
Habitus. **1**C. (C.) barclayi sp. n. **2**C. (C.) dimbyi sp. n. **3**C. (C.) fasciata sp. n. **4**C. (C.) lalae sp. n. **5**C. (C.) madagascarensis sp. n. **6**C. (C.) mamyi sp. n. **7**C. (C.) njakai sp. n. **8**C. (C.) obesa sp. n. **9**C. (C.) rindrai sp. n.

**Figures 10–18.**
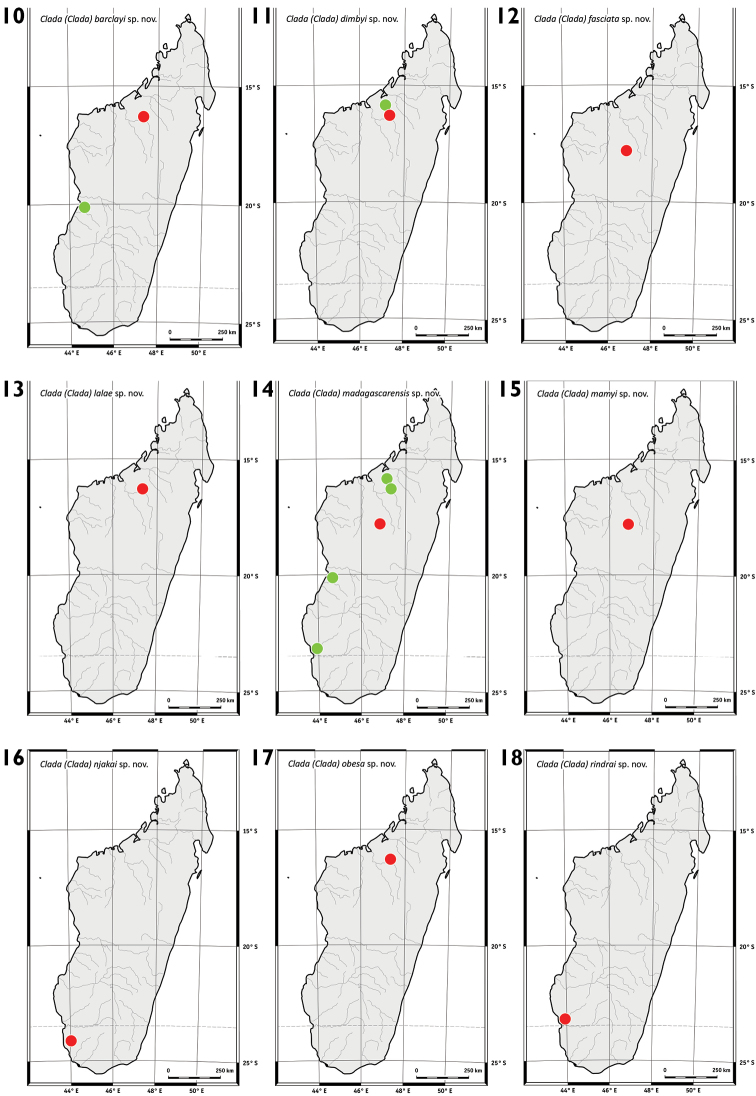
Maps of distribution. **10**C. (C.) barclayi sp. n. **11**C. (C.) dimbyi sp. n. **12**C. (C.) fasciata sp. n. **13**C. (C.) lalae sp. n. **14**C. (C.) madagascarensis sp. n. **15**C. (C.) mamyi sp. n. **16**C. (C.) njakai sp. n. **17**C. (C.) obesa sp. n. **18**C. (C.) rindrai sp. n.

**Figures 19–27.**
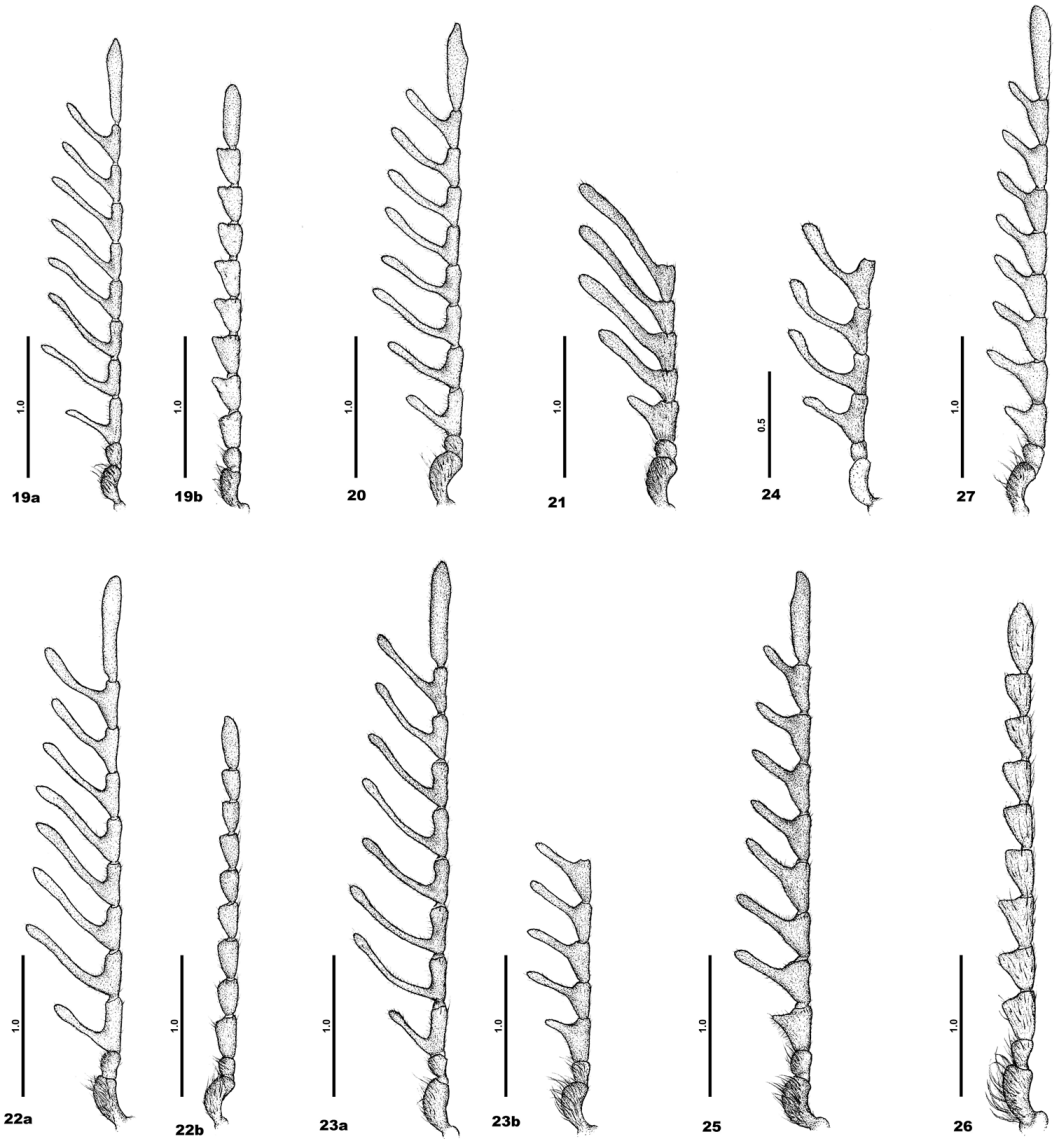
Antennae. **19**C. (C.) barclayi sp. n. – a male, b female **20**C. (C.) dimbyi sp. n. – male **21**C. (C.) fasciata sp. n. – male **22**C. (C.) lalae sp. n. – a male, b female **23**C. (C.) madagascarensis sp. n. – a male, b female **24**C. (C.) mamyi sp. n. – male **25**C. (C.) njakai sp. n. – male **26**C. (C.) obesa sp. n. – female **27**C. (C.) rindrai sp. n. – male.

**Figures 28–35.**
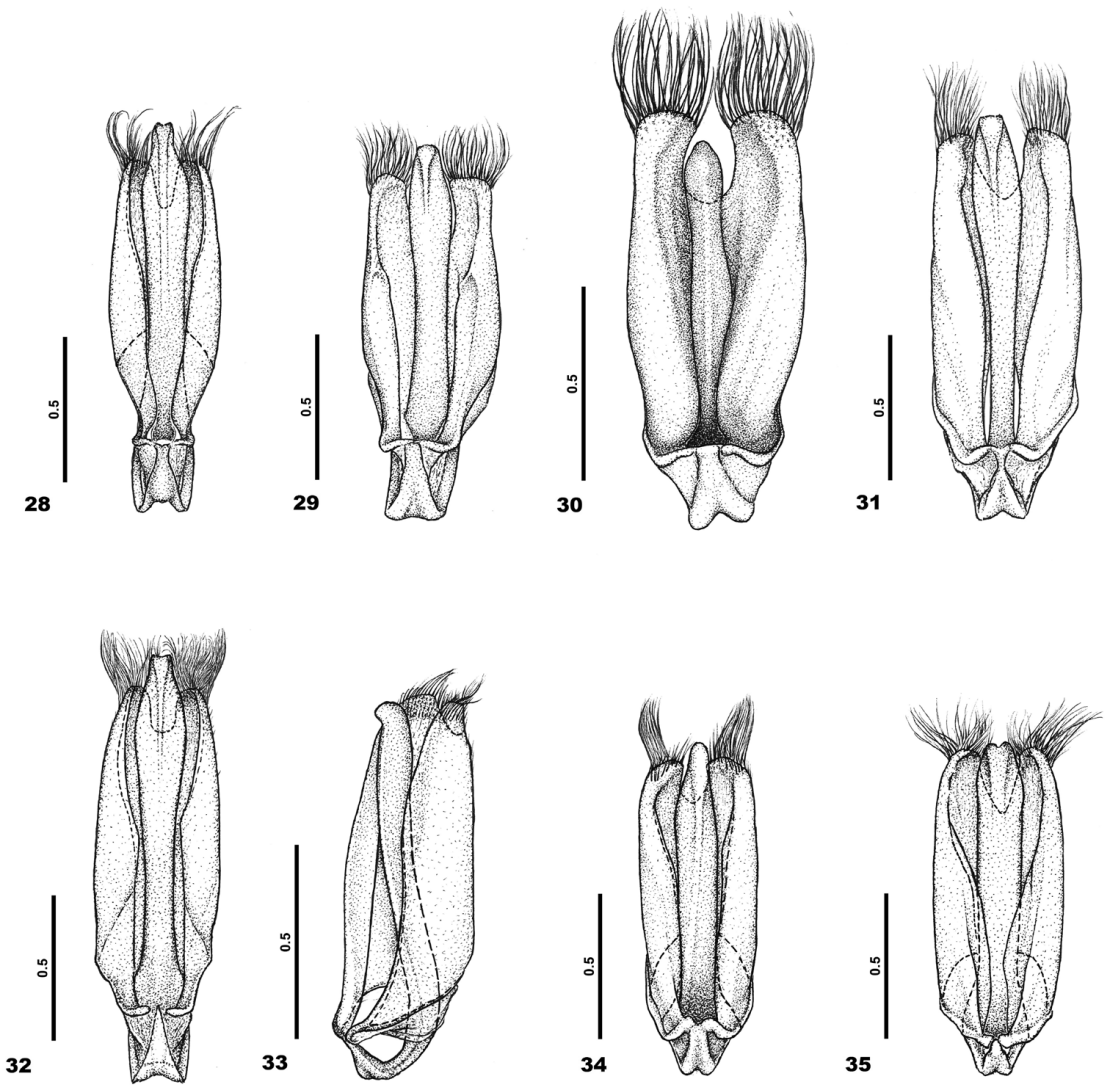
Aedeagus in dorsal viev. **28**C. (C.) barclayi sp. n. **29**C. (C.) dimbyi sp. n. **30**C. (C.) fasciata sp. n. **31**C. (C.) lalae sp. n. **32**C. (C.) madagascarensis sp. n. **33**C. (C.) mamyi sp. n. **34**C. (C.) njakai sp. n. **35**C. (C.) rindrai sp. n.

Figs 1 , 10 , 19a, b , 28

#### Type material.

**Holotype male**: Madagascar, Mahajanga prov., Ampatika env., Mahajamba riv., 17.–19.xi.1995, I. Jeniš lgt. (FGMRI). **Paratype (1)**: 1 female, Madagascar, Morondava prov., Maronfandilia, 4.–5.xii.1995, J. Stolarczyk lgt. (FGMRI).

#### Differential diagnosis.

This species is similar to C. (C.) humeralis Pic, 1926, but differs by lighter colour of the elytra and missing lighter humeri. Fully differs by shape of the aedeagus. Fully differs by shape of the aedeagus. For differences from other Madagascan species, see key.

#### Description.

Male (holotype). Elongate-elliptical, transversally convex. Body length 5.8 mm, maximum width 2.2 mm (Figure 1). Ratio length:width of elytra 1.7. Body light brown, also antennae, maxillary and labial palpi and legs, only pronotum and head darker. Pubescence yellowish white.

*Head* matt shiny, with double punctation – first coarse, dense, umbilicate, distance between punctures approximately the same as their diameter; second is very fine, punctures almost touching. Pubescence recumbent or semi-erect, long, inclined more or less forwards. Anterior part of head with shallow deepening. Clypeus with shallow transverse depression. Eyes large, globular with short erect sparse pubescence. Frons 1.6 times as wide as diameter of eye, from dorsal view. Antennae consisting of eleven antennomeres, 3^rd^ to 10^th^ pectinate (Figure 19a). First antennomere robust, twice as long as wide; second smallest, one-half as long as first, almost as wide as long. 3^rd^ 1.4 times as wide as long. 4^th^ to 8^th^ 2.1 as wide as long; 9^th^ and 10^th^ 1.7 times as wide as long. Apical antennomere longest, oblong oval, 5 times as long as wide. All antennomeres on margin with short erect dense setae. Apical maxillary palpomere long, spindle shaped.

*Pronotum* convex, matt shiny, rounded, transverse (ratio length:width of pronotum 0.8); widest on one half, but only slightly. Base of pronotum finely bordered. Middle of pronotum at base with small, blunt swelling, posteriorly slightly sharpened. Surface of pronotum with double punctation: one coarse, dense, umbilicate, distance between punctures approximately one-half their diameter; other one is very fine, punctures almost touching. Pubescence short, sparse, recumbent, inclined more or less forwards.

*Scutellum* almost triangular, narrow, 1.4 times as long as wide, dense recumbent pubescence, inclined backwards.

*Elytra* oval, transversally convex, shiny, with distinct humeri. Each elytron with five fine costae, almost invisible, but apex more distinct. Surface of elytra with double punctation: one coarse, dense, umbilicate, distance between punctures approximately the same as their diameter; the other one is very fine, punctures almost touching. Pubescence relatively sparse, recumbent or semi-erect, inclined backwards. Posterior margin of each elytron with approximately 25 small teeth.

Legs stout, with short and dense recumbent pubescence. All tarsi robust, same length as tibia. 1^st^ metatarsomere as long as 2^nd^ and 3^rd^ together, same width, slightly emarginate on top, 4^th^ slightly shorter than previous, more emarginate, almost to 2/3 of their length. 5^th^ same length as 3^rd^ and 4^th^ together, rectangular, wider on the top, with two large claws, without teeth.

For *aedeagus* see Figure 28.

Female. Habitually the same as male, only antennae serrate (Figure 19b). 1^st^ antennomere robust with dense long erect hairs. 2^nd^ small, as wide as 1^st^, half as long as previous, as long as wide. Antennomeres 3^th^ to 10^th^ serrate. 3^rd^ and 4^th^ twice longer than wide; 5^th^ 2.3 longer than wide; 6^th^ twice longer than wide; 7^th^ 1.7 times longer than wide; 8^th^ to 10^th^ twice longer than wide. Apical antennomere longest, oblong oval, 3.3 times longer than wide. Body length 6.8 mm, maximum width 2.9 mm. Ratio length:width of elytra 1.8.

#### Name derivation.

Patronym, dedicated to our friend and colleague Maxwell VL Barclay (Natural History Museum, London).

#### Biology.

Unknown.

#### Distribution.

This species is found in the western part of Madagascar (Figure 10).

### Clada (Clada) dimbyi sp. n.

Taxon classificationAnimaliaColeopteraPtinidae

http://zoobank.org/1591559C-96BE-4553-B410-39F33E8C2167

Figs 2 , 11 , 20 , 29

#### Type material.

**Holotype male**: Madagascar, Mahajanga prov., Mahajamba riv., Ampatika env., 17.–19.xi.1995, I. Jeniš lgt. (FGMRI). **Paratype(1)**: 1 male, Madagascar, Mahajanga prov., Ambodimanga, Ankolia riv., 14.–15.xi.1995, J. Stolarczyk lgt. (FGMRI).

#### Differential diagnosis.

The species is similar to C. (C.) humeralis Pic, 1926, but differs by the lighter colour of the elytra and absence of lighter coloured humeri. Fully differs by shape of the aedeagus. Fully differs by shape of the aedeagus. For differences from other Madagascan species, see key.

#### Description.

Male (holotype). Elongate-elliptical, transversally convex. Body length 5.9 mm, maximum width 2.9 mm (Figure 2). Ratio length:width of elytra 1.6. Body light brown, head and pronotum brown, antennae and legs partly darker. Pubescence white.

*Head* matt-shiny, with double punctation – one coarse, dense, umbilicate, distance between punctures approximately the same as their diameter; other is very fine, punctures almost touching. Pubescence recumbent or semi-erect, long, mostly inclined forwards, partly to centre of head, on vertex backwards. Clypeus with shallow, transverse depression. Eyes large, globular with short erect sparse pubescence. Frons twice as wide as diameters of eye, in dorsal view. Antennae consisting of eleven antennomeres; 3^rd^ to 10^th^ pectinate (Figure 20). 1^st^ antennomere robust, twice as long as wide; 2^nd^ smallest, only 1/3 as long as 1^st^, as long as wide, the same width as 1^st^. 3^rd^ 1.3 times as wide as long; 4^th^ and 5^th^ 2.1 times as wide as long; 6^th^, 7^th^ and 9^th^ 1.9 times as wide as long; 8^th^ twice wider as long; 10^th^ 1.6 times as wide as long. Apical antennomere longest, oblong oval, 5.7 times as long as wide. All antennomeres with short recumbent pubescence, only 1^st^ and 2^nd^ with a few long semi-erect setae. Apical maxillary palpomere long, spindle shaped.

*Pronotum* convex, matt-shiny, transverse (ratio length:width of pronotum 0.8); widest in posterior 2/3. Base of pronotum finely bordered. Middle of pronotum at base with a small blunt swelling, posteriorly slightly sharpened. Surface of pronotum with coarse, dense, umbilicate punctation, distance between punctures smaller than their diameter. Pubescence long, sparse, recumbent, inclined more or less forwards.

*Scutellum* large, longitudinally trapezoidal, 1.2 times as long as wide, densely recumbent pubescence, inclined backwards, surface shinning with fine, dense punctures.

*Elytra* oval, transversally convex, shiny, with distinct humeri. Each elytron with five very fine costae. Surface of elytra irregularly punctated with punctures of different diameters, coarse, dense, umbilicate. Pubescence relatively sparse, recumbent, on sides also semi-erect, inclined backwards. Posterior margin of each elytron with approximately 25 very small teeth.

*Legs* stout, with short and dense recumbent pubescence. Mesotibia on the apex with short forked projection. All tarsi robust, the same length as tibia. 1^st^ metatarsomere as long as 2^nd^ and 3^rd^ together, the same width, slightly emarginate on top, 4^th^ slightly shorter than previous, more emarginate, almost to 2/3 of their length. 5^th^ the same length as 3^rd^ and 4^th^ together, rectangular, wider on the top, with two large claws, without teeth.

For *aedeagus* see Figure 29.

Female. Unknown.

#### Variability.

Without visible variability.

#### Name derivation.

Patronymic, dedicated to Dr Dimby Raharinjanahary from Madagascar National Parks, Antananarivo (Chargé des Bases de données de suivibiodiversité et recherche).

#### Biology.

Unknown.

#### Distribution.

This species is found in the northwestern part of Madagascar (Figure 11).

### Clada (Clada) fasciata sp. n.

Taxon classificationAnimaliaColeopteraPtinidae

http://zoobank.org/B411F2DE-4892-46F4-842E-6B0E1A9FD67D

Figs 3 , 12 , 21 , 30

#### Type material.

**Holotype male**: Madagascar, Antananarivo prov., Manankazo env., 15.–17.xii.1996, I. Jeniš lgt. (FGMRI).

#### Differential diagnosis.

The species is similar to C. (C.) lineatipennis Pic, 1926, which has black coloured elytra, and C. (C.) costipennis Kolbe, 1897, C. (C.) flabellicornis Pic, 1936 and C. (C.) multistriata Pic, 1952 whose males have pectinated antennae. Differs also by shape of the aedeagus. For differences from other Madagascan species, see key.

#### Description.

Male (holotype). Elongate-elliptical, transversally convex. Body length 6.0 mm, maximum width 2.6 mm (Figure 3). Ratio length:width of elytra 1.7. Body, including antennae, maxillary and labial palpi and legs, brown. Only pronotum piceous brown. Pubescence white.

*Head* shiny, with double punctation – first coarse, dense, umbilicate, distance between punctures approximately the same as their diameter; other very fine, punctures almost touching. Pubescence more or less recumbent, long, inclined backwards; on vertex inclined backwards. Clypeus with transverse depression. Eyes large, globular with short erect sparse pubescence. Frons 3 times as wide as diameters of the eye, from dorsal view. Antennae probably consisting of eleven antennomeres (they are damaged, only 7 antennomeres remain), from 4^th^ pectinate (Figure 21). 1^st^ antennomere robust, twice as long as wide; 2^nd^ as wide as 1^st^, 0.3 as long as 1^st^, 0.8 times as wide as long. 3^rd^ strongly serrate, 1.1 times as wide as long. 4^th^ and 5^th^ 2.5 times as wide as long. 6^th^ 3 times as wide as long; 7^th^ 2.7 times as wide as long. All antennomeres with very short recumbent dense pubescence, 1^st^ also with sparse long semi-erect setae. Apical maxillary palpomere long, slim, spindle shaped.

*Pronotum* convex, matt-shiny, transverse (ratio length:width of pronotum 0.7); widest in middle. Middle of the pronotum with blunt small swelling. Surface of pronotum with coarse, dense, umbilicate punctuation; punctures almost touching. Pubescence long, dense, semi-erect, inclined more or less from middle of pronotum to all sides.

*Scutellum* large, triangular, narrow, 1.2 times as long as wide, very densely recumbent pubescence, inclined backwards, surface shining, finely punctated; punctures almost touching.

*Elytra* oval, transversally convex, shining, humeri almost absent. Each elytron with six fine costae, covered with white recumbent dense pubescence, inclined backwards and from sides of costa to their centre. Surface of elytra with double punctation – one coarse, dense, umbilicate, almost touching; other is very fine, punctures also almost touching. Pubescence between stripes relatively sparse, recumbent or semi-erect, inclined backwards. Posterior margin of each elytron with approximately 20 small teeth, almost invisible.

*Legs* stout, with long, dense, recumbent pubescence. All tarsi robust, same length as tibia. 1^st^ metatarsomere as long as 2^nd^ and 3^rd^ together, same width, slightly emarginate on top, 4^th^ slightly shorter than previous, more emarginate, almost to 2/3 of their length. 5^th^ the same length as 3^rd^ and 4^th^ together, rectangular, wider on the top, with two large claws, without teeth.

For *aedeagus* see Figure 30.

Female. Unknown.

#### Name derivation.

Derived from the rows of dense recumbent hairs on elytra, from Latin word *fascia*, meaning stripe.

#### Biology.

Unknown.

#### Distribution.

This species is found in the central part of Madagascar (Figure 12).

### Clada (Clada) lalae sp. n.

Taxon classificationAnimaliaColeopteraPtinidae

http://zoobank.org/F199462F-C7C5-4735-8C77-618D7CCF3053

Figs 4 , 13 , 22a, b , 31

#### Type material.

**Holotype male**: Madagascar, Mahajanga prov., Mahajamba riv., Ampatika env., 17.–19.xi.1995, I. Jeniš lgt. (FGMRI). **Paratypes (5)**: 2 males, 1 female, the same data as holotype; 2 males, Madagascar, Mahajanga prov., Ampatika env., 17.–20.xii.1995, J. Stolarczyk lgt. (FGMRI).

#### Differential diagnosis.

This species is similar to C. (C.) humeralis Pic, 1926, but differs by the lighter colour of the elytra. Fully differs by shape of the aedeagus. For differences from other Madagascan species, see key.

#### Description.

Male (holotype). Elongate-elliptical, transversally convex. Body length 7.0 mm, maximum width 2.9 mm (Figure 4). Ratio length:width of elytra 1.7. Body dark brown; antennae, maxillary and labial palpi and legs lighter. Pubescence white.

*Head* matt, clypeus shiny, with coarse, dense, umbilicate punctation; distance between punctures approximately the same as their diameter. Pubescence recumbent, long, dense, inclined mostly forwards. Clypeus with shallow transverse depression. Eyes large, globular with long erect sparse pubescence. Frons 2.1 times as wide as diameter of eye, from dorsal view. Antennae consisting of eleven antennomeres, 3^rd^ to 10^th^pectinate (Figure 22a). 1^st^ antennomere robust, three times as long as wide; 2^nd^ smallest, twice shorter than 1^st^, as long as wide, almost same width as 1^st^. 3^rd^ 0.8 times shorter than wide; 4^th^and 6^th^ to 8^th^ 0.5 times shorter than wide; 9^th^ and 10^th^ 0.6 times shorter than wide and the 10^th^ 0.7 times shorter than wide. Apical antennomere longest, oblong oval, 5 times as long as wide. All antennomeres with short recumbent pubescence, only 1^st^ and 2^nd^ with a few long semi-erect setae. Apical maxillary palpomere long, spindle shaped.

*Pronotum* convex, matt, transverse (ratio length:width of pronotum 0.7); widest in posterior 2/3. Base of pronotum finely bordered. Middle of pronotum in posterior part with blunt small swelling, posteriorly slightly sharpened. Surface of pronotum with coarse, dense, umbilicate, distance between punctures the same as their diameter. Pubescence short, sparse, recumbent, inclined more or less forwards, in posterior part of pronotum backwards.

*Scutellum* large, longitudinally rectangular, 1.3 times as long as wide, densely recumbent pubescence, inclined backwards, surface shinning with fine dense puncture.

*Elytra* oval, transversally convex, shiny, with distinct humeri. Each elytron with fine costae. Surface of elytra irregularly wrinkled, with double punctation – one coarse, dense, umbilicate, punctures almost touching; other is very fine, punctures also almost touching. Pubescence relatively sparse, recumbent, on sides also semi-erect and sporadically also erect, inclined backwards. Posterior margin of each elytron with approximately 25 very small teeth.

*Legs* stout, with short and dense recumbent pubescence. All tarsi slim, slightly shorter than tibia. 1^st^ metatarsomere as long as 2^nd^ and 3^rd^ together, and same length as 5^th^. 2^nd^ the same length as 3^rd^ and 4^th^ together. 4^th^ emarginate approximately to ½ of their length. 5^th^ long and robust with two large claws, without teeth.

For *aedeagus* see Figure 31.

Female. Habitually the same as male, only antennae serrate (Figure 22b). Body length 8.1 mm, maximum width 3.2 mm.

#### Variability.

Body length 5.4–8.1 mm, maximum width 2.2–3.2 mm.

#### Name derivation.

Patronym, dedicated to Dr Lala Harivelo Ravaomanarivo Raveloson (University of Antananarivo, Faculty of Sciences, Department of Entomology).

#### Biology.

Unknown.

#### Distribution.

This species is found in the northwestern part of Madagascar (Figure 13).

### Clada (Clada) madagascarensis sp. n.

Taxon classificationAnimaliaColeopteraPtinidae

http://zoobank.org/32F329CB-F712-49DB-80E6-F28B4EE00B18

Figs 5 , 14 , 23a, b , 32

#### Type material.

**Holotype male**: Madagascar, Mahajanga distr., Ampatika env., 17.–20.xi.1995, J. Stolarczyk lgt. (FGMRI). **Paratypes (21)**: 4 males and 1 female, the same data as holotype (FGMRI); 10 males, Madagascar, Morondava distr., Kirindy, 23.–25.xi.1997, J. Stolarczyk lgt. (FGMRI 4 ex., LBVC 2 ex., MTDC 2 ex., NHMUK 2 ex.); 1 male: Madagascar, Mahajanga distr., Ambodimanga env., 14.–16.xi.1995, J. Stolarczyk lgt. (FGMRI); 2 males: Madagascar, Morondava distr., Maronfandilia, 4.v.1995, J. Stolarczyk lgt. (FGMRI); 2 male: Madagascar, Mahajanga prov., Mahajamba riv., Ampatika env., 17.–19.xi.1995, I. Jeniš lgt. (FGMRI); 1 female: Madagascar, Toliara env., 23.–27.xi.1996, J. Stolarczyk lgt. (FGMRI).

#### Differential diagnosis.

This species is similar to C. (C.) humeralis Pic, 1926, but differs by the lighter colour of the elytra and missing lighter humeri. Both sexes have pectinate antennae, while the female of C. (C.) humeralis Pic, 1926 has serrate antennae. Fully differs by shape of the aedeagus. For differences from other Madagascan species, see key.

#### Description.

Male (holotype). Elongate-elliptical, transversally convex. Body length 6.0 mm, maximum width 2.5 mm (Figure 5). Ratio length:width of elytra 1.6. Whole body brown, only antennae, palp slightly lighter and pronotum slightly darker. Pubescence white.

*Head* shiny, with double punctation – one coarse, dense, umbilicate, distance between punctures approximately the same as their diameter, sometimes almost touching; other one is very fine, punctures almost touching. Pubescence recumbent or semi-erect, long, inclined mostly forwards. Clypeus with shallow transverse depression. Eyes large, globular with short erect sparse pubescence. Frons twice as wide as diameters of the eye, from dorsal view. Antennae consisting of eleven antennomeres, 4^th^ to 10^th^pectinate (Figure 23a). 1^st^ antennomere robust, twice as long as wide; 2^nd^ smallest, only 1/3 long as 1^st^, as wide as long, same width as the 1^st^. 3^rd^ serrate, as long as wide; 4^th^ to 8^th^ twice long as wide; 9^th^ 1.7 times as wide as long; 10^th^ 1.5 times as wide as long. Apical antennomere longest, oblong oval, 6.6 times as long as wide. All antennomeres with short recumbent pubescence, only 1^st^ with a few long semi-erect setae. Apical maxillary palpomere long, spindle shaped.

*Pronotum* convex, matt-shiny, transverse (ratio length:width of pronotum 0.7); widest in middle. Base of pronotum finely bordered. Middle of pronotum at base with blunt small swelling, posteriorly slightly sharpened. Surface of pronotum with double punctation – one coarse, dense, umbilicate, distance between punctures approximately the same as their diameter; other one is very fine, punctures almost touching. Pubescence long, sparse, recumbent, inclined more or less to middle of pronotum.

*Scutellum* large, longitudinally trapezoidal, narrow, almost as long as wide, densely recumbent pubescence, inclined backwards, surface almost invisible.

*Elytra* oval, transversally convex, shining, with distinct humeri. Each elytron with five very fine costae, more distinct on second half of elytron. Surface of elytra irregular punctated, puncture coarse, dense, umbilicate. Pubescence relatively sparse, recumbent, inclined backwards. Posterior margin of each elytron with approximately 25 very small teeth.

*Legs* stout, with short and dense recumbent pubescence. All tarsi robust, same length as tibia. 1^st^ metatarsomere as long as 2^nd^ and 3^rd^ together, same width, slightly emarginate on top, 4^th^ slightly shorter than previous, more emarginate, almost to 2/3 of their length. 5^th^ is same length as 3^rd^ and 4^th^ together, rectangular, wider on the top, with two large claws, without teeth.

For *aedeagus* see Figure 32.

Female. Antennae less pectinate than in male (damaged, only six antennomeres remain – Figure 23b). Body length 5.7 mm, maximum width 2.1 mm.

#### Variability.

Body length 5.7–7.1 mm, maximum width 2.1–2.8 mm.

#### Name derivation.

Latin adjective, referring to the occurrence of the new species in Madagascar.

#### Biology.

Unknown.

#### Distribution.

This species is found in the western part of Madagascar (Figure 14).

### Clada (Clada) mamyi sp. n.

Taxon classificationAnimaliaColeopteraPtinidae

http://zoobank.org/C07FB3A7-6981-4570-920C-220305AC455F

Figs 6 , 15 , 24 , 33

#### Type material.

**Holotype male**: Madagascar, Antananarivo prov., Manankazo env., 15.–17.xii.1996, I. Jeniš lgt. (FGMRI).

#### Differential diagnosis.

Differs from other species of this genus from sub-saharan and southern African regions by a lack of elytral costae. Fully differs by shape of the aedeagus. For differences from other Madagascan species, see key.

#### Description.

Male (holotype). Elongate-elliptical, transversally convex. Body length 3.9 mm, maximum width 1.6 mm (Figure 6). Ratio length:width of elytra 1.7. Body brown, head and pronotum darker, antennae lighter. Pubescence white.

*Head* shining, with coarse, dense, umbilicate punctated, distance between punctures approximately the same as their diameter. Pubescence recumbent, short, sparse, inclined mostly forwards. Clypeus with shallow transverse depression. Eyes large, globular with very short erect sparse pubescence, almost invisible. Frons 1.3 times as wide as diameter of the eye, from dorsal view. Antennae probably consisting of eleven antennomeres (they are damaged, only 6 antennomeres remain – Figure 24), 3^rd^ to 6^th^pectinate. 1^st^ antennomere robust, three times as long as wide; 2^nd^ smallest, 3 times shorter than 1^st^, as long as wide, same width as 1^st^. 3^rd^ 1.2 times wider as long; 4^th^ 1.3 times wider than long; 5^th^ and 6^th^ 2.2 times wider than long. Other antennomeres are slightly damages or missing. All antennomeres without pubescence. Apical maxillary palpomere short, spindle shaped.

*Pronotum* convex, matt-shiny, transverse (ratio length:width of pronotum 0.6); widest in middle. Base of pronotum finely bordered. Pronotum without swelling. Surface of pronotum with coarse, dense, umbilicate, distance between punctures smaller than their diameter. Pubescence long, sparse, recumbent, inclined more or less forwards.

*Scutellum* large, longitudinally almost rectangular, 1.1 times as long as wide, densely recumbent pubescence, inclined backwards, surface shinning with fine dense puncture.

*Elytra* oval, transversally convex, shining, humeri almost indistinct. Each elytron with only very fine quasi-costae. Surface of elytra with double punctation – one coarse, dense, umbilicate, punctures almost touching; other one is very fine, punctures also almost touching. Pubescence relatively sparse, recumbent, on sides also semi-erect, inclined backwards. Posterior margin of each elytron with approximately 25 very small teeth.

*Legs* stout, with short and dense recumbent pubescence. All tarsi slim, 1.2 as long as tibia. 1^st^ metatarsomere as long as 2^nd^ to 4^th^ together, and same length as 5^th^. 2^nd^ is same length as 3^rd^ and 4^th^ together. 4^th^ only slightly emarginate. 5^th^ long and slim with long slim claws, without teeth.

For *aedeagus* see Figure 33.

Female. Unknown.

#### Name derivation.

Patronym, dedicated to Dr Mamy A Rakotoarijaona from Madagascar National Parks, Antananarivo (Directeur des Opérations).

#### Biology.

Unknown.

#### Distribution.

This species is found in the central part of Madagascar (Figure 15).

### Clada (Clada) njakai sp. n.

Taxon classificationAnimaliaColeopteraPtinidae

http://zoobank.org/D3CD1585-C840-41A0-9290-E8FFCD375206

Figs 7 , 16 , 25 , 34

#### Type material.

**Holotype male**: Madagascar, Toliara prov., Tsimanampetsotsa N. P., Mitoho camp, 24°02.898'S, 43°45.138'E, 10 m a. s. l., 12.–13.i.2014, M. Trýzna leg. **Paratypes (15)**: 1 male: the same data as holotype; 13 males: Madagascar, Toliara prov., Tsimanampetsotsa N. P., Andranovao camp, 24°01.505'S, 43°44.306'E, 15 m a. s. l., 14.–15.i.2014, M. Trýzna leg. (FGMRI 7 ex., LBVC 2 ex., MTDC 2 ex., NHMUK 2 ex.).

#### Differential diagnosis.

This species is similar to C. (C.) humeralis Pic, 1926, but differs by the lighter colour of the elytra and missing lighter humeri. Fully differs by shape of the aedeagus. For differences from other Madagascan species, see key.

#### Description.

Male (holotype). Elongate-elliptical, transversally convex. Body length 5.3 mm, maximum width 2.1 mm (Figure 7). Ratio length:width of elytra 1.8. Whole body dark brown, only antennae, maxillary and labial palpi and humeri on elytra moderately lighter. Pubescence yellowish-white.

*Head* matt-shiny, with double punctation – one coarse, dense, umbilicate, distance between punctures approximately the same as their diameter; other one very fine, punctures almost touching. Pubescence recumbent or semi-erect, short, inclined backwards; on sides of head semi-erect and long, inclined forwards. Clypeus with shallow transverse depression. Eyes large, globular with short erect sparse pubescence. Frons twice as wide as diameters of eye, from dorsal view. Antennae consisting of eleven antennomeres, 4^th^ to 10^th^ pectinate (Figure 25). 1^st^antennomere robust, twice as long as wide; 2^nd^ smallest, only one-half length of 1^st^, as long as wide, slightly narrower than 1^st^. 3^rd^ serrate, as long as wide; 4^th^ and 5^th^ 1.8 times wider than long; 6^th^ and 7^th^ 1.5 times wider than long; the 8^th^ and 9^th^ 1.3 times wider as long; and 10^th^ 1.1 times as wide as long. Apical antennomere is longest, oblong oval, 4.3 times as long as wide. All antennomeres on margin with short erect dense setae. Apical maxillary palpomere long, spindle shaped.

*Pronotum* convex, matt-shiny, rounded, transverse (ratio length:width of pronotum 0.7); widest at 2/3 posteriorly. Base of pronotum finely bordered. Middle of pronotum at base with blunt small swlling, posteriorly slightly sharpened. Surface of pronotum with double punctation, one coarse, dense, umbilicate, distance between punctures approximately the same as their diameter; other one is very fine, punctures almost touching. Pubescence long, sparse, recumbent, inclined more or less to middle of pronotum.

*Scutellum* triangular, narrow, 1.3 times as long as wide, very densely recumbent pubescence, inclined backwards, surface is not visible.

*Elytra* oval, transversally convex, shining, with distinct humeri. Each elytron with five fine costae, almost invisible, but apex more distinct. Surface of elytra with double punctation, one coarse, dense, umbilicate, distance between punctures approximately the same as their diameter; other one is very fine, punctures almost touching. Pubescence relatively sparse, recumbent or semi-erect, inclined backwards. Posterior margin of each elytron with approximately 25 small teeth.

*Legs* stout, with short and dense recumbent pubescence. All tarsi robust, same length as tibia. 1^st^ metatarsomere as long as 2^nd^ and 3^rd^ together, same width, slightly emarginate on top, 4^th^ slightly shorter than previous, more emarginate, almost to 2/3 of their length. 5^th^ is same length as 3^rd^ and 4^th^ together, rectangular, wider on the top, with two large claws, without teeth.

For *aedeagus* see Figure 34.

Female. Unknown.

#### Variability.

Body length 4.7–7.1 mm, maximum width 1.8–2.8 mm.

#### Name derivation.

Patronym, dedicated to Adolphe Randrianjaka (University of Antananarivo, Faculty of Sciences, Department of Entomology), whom we called Njaka.

#### Biology.

Unknown. All specimens were collected at light.

#### Distribution.

This species is found in the southwestern part of Madagascar (Figure 16).

### Clada (Clada) obesa sp. n.

Taxon classificationAnimaliaColeopteraPtinidae

http://zoobank.org/D6AB5731-53E8-4709-9FDB-61614AEEB6F9

Figs 8 , 17 , 26

#### Type material.

**Holotype female**: Madagascar, Mahajanga prov., Ampatika env., Mahajamba riv., 10.–12.xii.1996, I. Jeniš lgt. (FGMRI).

#### Differential diagnosis.

Differs from other African species by the shape of the body, which is very arched. For differences from other Madagascan species, see key.

#### Description.

Female (holotype). Short, elongate-elliptical, extremely strongly transversally convex (any other species from genus *Clada* Pascoe, 1887 is not so convex). Body length 8.0 mm, maximum width 4.4 mm (Figure 8). Ratio length:width of elytra 1.6. Body dark brown, pronotum piceous-black, legs dark brown, antennae and maxillary and labial palpi lighter, brown. Pubescence yellowish white.

*Head* matt; dense, coarse, umbilicate punctation, with long recumbent or semi-erect dense pubescence, with sparse long erect setae, inclined more or less forwards, only on vertex partly inclined to middle or backwards. Clypeus with deep transversal furrow, frons flattened. Eyes large, globular with short erect sparse brown pubescence. Frons wide, 2.9 times as wide as diameter of eye (from dorsal view). Antennae consisting of eleven antennomeres, serrate (Figure 26). 1^st^ antennomere robust, twice as long as wide, with dense long erect hairs; 2^nd^ small, as wide as 1^st^, only one-half of their length, as long as wide. 3^rd^ slightly serrate, 1.5 times as long as wide. Antennomeres 4^th^ to 10^th^ serrate; 4^th^ 1.1 times as long as wide, 5^th^ 1.5 times as long as wide; 6^th^ 1.3 times as long as wide, 7^th^ to 10^th^ 1.6 times as long as wide, 11^th^ oblong oval, 2.6 times as long as wide. Apical maxillary palpomere long, spindle shaped.

*Pronotum* convex, matt shining, transverse (ratio length:width of pronotum 0.7), widest in last third. Base of pronotum finely bordered. Middle of pronotum at base with high blunt swelling, on their sides shallow, almost invisible rounded depression. Surface of pronotum coarsely, densely, umbilicate punctate, distance between punctures smaller than their diameter, almost touching. Pubescence short, recumbent or semi-erect, inclined largely backwards, on sides inclined obliquely backward, on anterior margin inclined from sides to middle; from anterior margin to swelling in middle arranged to narrow strip.

*Scutellum* large, almost triangular (on top slightly rounded), 1.2 times as long as wide. Surface distinct with dense and coarse umbilicate punctation, with short, dense, recumbent pubescence inclined backwards.

*Elytra* short oval, transversally strongly convex, shining, with distinct humeri. Each elytron slightly irregular bent, with fifth costae, which are only slightly visible (especially from lateral view). Surface of elytra with double punctation. One very coarse, dense, umbilicate, irregular, diameter between punctures smaller than their diameter. Other one relatively fine, dense; punctures almost touching. Pubescence short, sparse, inclined backwards.

*Legs* stout, with short and dense recumbent pubescence. All tarsi robust, same length as tibia. 1^st^ metatarsomere as long as 2^nd^ and 3^rd^ together, same width, slightly emarginate on top, 4^th^ slightly shorter than previous, more emarginate, almost to 2/3 of their length. 5^th^ is same length as 3^rd^ and 4^th^ together, rectangular, wider on the top, with two large claws, without teeth.

Male. Unknown.

#### Name derivation.

Derived from the shape of body, from the Latin *obesus* for plump.

#### Biology.

Unknown.

#### Distribution.

This species is found in the northwestern part of Madagascar (Figure 17).

### Clada (Clada) rindrai sp. n.

Taxon classificationAnimaliaColeopteraPtinidae

http://zoobank.org/55124B57-580F-4909-A8BC-79414B5C1B23

Figs 9 , 18 , 27 , 35

#### Type material.

**Holotype male**: Madagascar, Toliara prov., Toliara env., 23.–27.xi.1996, J. Stolarczyk lgt. (FGMRI).

#### Differential diagnosis.

This species is similar to C. (C.) humeralis Pic, 1926, but differs by the lighter colour of the elytra and missing lighter humeri. Fully differs by shape of the aedeagus. For differences from other Madagascan species, see key.

#### Description.

Male (holotype). Elongate-elliptical, transversally convex. Body length 6.6 mm, maximum width 2.5 mm (Figure 9). Ratio length:width of elytra 1.5. Body brown, pronotum darker; antennae, maxillary and labial palpi and legs lighter. Pubescence yellowish white.

*Head* matt-shiny, with double punctation – one coarse, dense, umbilicate, distance between punctures approximately the same as their diameter; other one is very fine, almost invisible, punctures almost touching. Pubescence semi-erect, long, in anterior part inclined forwards, in posterior part inclined more or less backwards. Clypeus with shallow transverse depression, shiny. Eyes large, globular with long erect dense pubescence. Frons 2.7 times as wide as diameter of the eye, from dorsal view. Antennae consisting of eleven antennomeres, 4^th^ to 10^th^ pectinate (Figure 27). 1^st^ antennomere robust, 2.5 times as long as wide; 2^nd^ smallest, almost triangular, only one-half long as the 1^st^, as long as wide, slightly narrower as the 1^st^. The 3^rd^ strongly serrate, as long as wide, 1.8 times as width of 1^st^. 4^th^ to 9^th^ 1.4 times as wide as long; 10^th^ 0.9 times shorter as long. Apical antennomere is longest, oblong oval, 5.7 times as long as wide. All antennomeres on margin with short erect dense setae. Apical maxillary palpomere long, spindle shaped.

*Pronotum* convex, matt-shiny, rounded, transverse (ratio length:width of pronotum 0.8); widest at 2/3 posteriorly. Base of pronotum finely bordered. Middle of pronotum at base with blunt small swelling, posteriorly slightly sharpened. Surface of pronotum with double punctation – one coarse, dense, umbilicate; distance between punctures approximately one half of their diameter, some punctures almost touching; other one is very fine, punctures almost touching. Pubescence long, dense, semi-erect, inclined more or less to middle of pronotum, only on sides inclined to margin.

*Scutellum* triangular with blunt top, narrow, 1.8 times as long as wide, very sparse and short recumbent pubescence, inclined backwards.

*Elytra* oval, transversally convex, shiny, with distinct humeri. Each elytron with five fine costae, almost invisible, but apex more distinct. Surface of elytra with double punctation, one coarse, dense, umbilicate, distance between punctures approximately the same as their diameter; other one is very fine, punctures almost touching. Pubescence relatively sparse, recumbent partly also semi-erect, inclined backwards. Posterior margin of each elytron with approximately 25 small teeth.

Legs stout, with short and dense recumbent pubescence. All tarsi robust, same length as tibia. 1^st^ metatarsomere as long as 2^nd^ and 3^rd^ together, same width, slightly emarginate on top. 4^th^ slightly shorter than previous, more emarginate, almost to 2/3 of their length. 5^th^ is same length as 3^rd^ and 4^th^ together, rectangular, wider on the top, with two large claws, without teeth.

For *aedeagus* see Figure 35.

Female. Unknown

#### Name derivation.

Patronym, dedicated to Mr Rindra Andriamahefasoa (Chef de Volet Conservation et Recherche, Andasibe-Mantadia National Park).

#### Biology.

Unknown.

#### Distribution.

This species is found in the southwestern part of Madagascar (Figure 18).

### Key to *Clada* (s. str.) from Madagascar

**Table:** 

1	Body extremely strongly convex, large species (8 mm), quite differ by shape of body from other species of this genus; between swelling and posterior margin of pronotum wide flattened (only female holotype known)	**Clada (Clada) obesa sp. n.**
–	Body convex, smaller (maximum 7 mm), between swelling and posterior margin only very slim flattened (males)	**2**
2	Elytra with distinct longitudinal rows of dense recumbent hairs, aedeagus Fig. 30	**Clada (Clada) fasciata sp. n.**
–	Elytra without distinct rows of dense recumbent hairs	**3**
3	Elytra with very fine quasi costae or without distinct costae, eyes with very short hairs, aedeagus Fig. 33	**Clada (Clada) mamyi sp. n.**
–	Elytra with more or less distinct costae, eyes with distinct hairs	**4**
4	The 3^rd^antennomere serrate	**5**
–	The 3^rd^antennomere pectinate	**6**
5	Swelling on pronotum sharpened, aedeagus Fig. 34	**Clada (Clada) njakai sp. n.**
–	Swelling on pronotum blunt, aedeagus Fig. 35	**Clada (Clada) rindrai sp. n.**
6	Lateral projection of the 3^rd^antennomere shorter than the length of this antennomere, aedeagus Fig. 32	**Clada (Clada) madagascarensis sp. n.**
–	Lateral projection of the 3^rd^antennomere longer than the length of this antennomere	**7**
7	Elytra yellow-brown	**8**
–	Elytra dark brown, aedeagus Fig. 31	**Clada (Clada) lalae sp. n.**
8	The 2^nd^antennomere as long as wide, aedeagus Fig. 29	**Clada (Clada) dimbyi sp. n.**
–	The 2^nd^antennonere distinctly longer than wide, aedeagus Fig. 28	**Clada (Clada) barclayi sp. n.**

**Table 1.** Main distinguishing characters of species of the genus *Clada* (s. str.) from the Southern African Region and Madagascar.

Species	Antennae		Costae/rows of hairs	Colour of elytra	Figure of aedeagus
	male	female			
C. (C.) barclayi sp. n.	P	S	yes/no	light brown	Zahradník & Trýzna
C. (C.) basilewskyi Español, 1969	S		yes/no	dark brown	Español, 1969b
C. (C.) costipennis Kolbe, 1897	P		yes/yes	dark brown	Absent
C. (C.) dimbyi sp. n.	P		yes/no	light brown	Zahradník & Trýzna
C. (C.) fasciata sp. n.	S		yes/yes	Brown	Zahradník & Trýzna
C. (C.) flabellicornis Pic, 1936	P		yes/yes	Rusty	absent
C. (C.) granulata Español, 1972	S		yes/no	Black	Español, 1972
C. (C.) humeralis Pic, 1926	P	S	yes/no	black (piceous)	absent
C. (C.) lalae sp. n.	P	S	yes/no	dark brown	Zahradník & Trýzna
C. (C.) laticollis Pic, 1947			yes/no	black-piceous (immature light brown)	absent
C. (C.) lineatipennis Pic, 1926			yes/yes	black (piceous)	absent
C. (C.) longicornis Pic, 1934	F	F	yes/no	Rusty	absent
C. (C.) madagascarensis sp. n.	P	P	yes/no	Brown	Zahradník & Trýzna
C. (C.) mamyi sp. n.	P		no/no	Brown	Zahradník & Trýzna
C. (C.) multistriata Pic, 1952	P		yes/yes	black (piceous)	absent
C. (C.) njakai sp. n.	P		yes/no	dark brown	Zahradník & Trýzna
C. (C.) obesa sp. n.		S	yes/no	dark brown	absent
C. (C.) rindrai sp. n.	P		yes/no	Brown	Zahradník & Trýzna
C. (C.) rugosa Pic, 1915	P		yes/no	Rusty	no
C. (C.) waterhousei Pascoe, 1887	P		yes/no	rusty or black	no

Abbreviations: F-filiform; P-pectinate; S-serrate

## Supplementary Material

XML Treatment for Clada (Clada) barclayi

XML Treatment for Clada (Clada) dimbyi

XML Treatment for Clada (Clada) fasciata

XML Treatment for Clada (Clada) lalae

XML Treatment for Clada (Clada) madagascarensis

XML Treatment for Clada (Clada) mamyi

XML Treatment for Clada (Clada) njakai

XML Treatment for Clada (Clada) obesa

XML Treatment for Clada (Clada) rindrai

## References

[B1] Bellés X (1987) Xylodes (Diegous) excavaticollis n. sp. de Madagascar (Col., Ptinidae). Bulletin de la Société Entomologique de France 102: 29–31.

[B2] Bellés X (1991) Insectes Coléoptères Ptinidae. Faune de Madagascar. 77. Muséum National d’Histoire Naturelle Paris. Faune de Madagascar 77: 1–122.

[B3] Español F (1969a) Notas sobre anóbidos (Col.). XXXVII.-XXXIX. [XXXVII. Más datos sobre los *Stagetus* del África tropical; XXXVIII. Dos nuevos *Stagetus* del Asia paleártica; XXXIX. Un nuevo Anobiinae de Madagascar]. Publicaciones del Instituto de Biologia Aplicada 46: 49–64.

[B4] Español F (1969b) Notas sobre anóbidos (Col.). XLI. Contribución al conocimiento de las *Clada* Pasc. Africanas. Miscelánea Zoológica (Barcelona) 2(4): 39–46.

[B5] Español F (1972) Notas sobre anóbidos (Col.). LVI.-LVIII. [LVI. Los *Xyletinus* Latr. de Madagascar; LVII. Descripción de dos nuevas especies del’Africa meridional; LVIII. Sobre un nuevo género de Xyletininae de las islas Canarias]. Publicaciones del Instituto de Biologia Aplicada 52: 49–65.

[B6] Kolbe HJ (1897) Coleopteren. Die Käfer Deutsch-Ost-Afrikas. Berlin: D. Reimer, 367 pp. 10.5962/bhl.title.53492

[B7] Pascoe FLS (1887) Notes on Coleoptera, with Descriptions of new Genera and Species. Part VI. The Annals and Magazine of Natural History (5)20: 8–20 + 1 pl.

[B8] Philips TK (2005) *Acanthaptinustriplehorni*, a new genus and species of spider beetle (Coleoptera: Ptinidae) from Madagascar. Annales Zoologici 55: 483–587.

[B9] Pic M (1896) Ptinidae recueillis à Madagascar par M. Charles Alluaud 1893 [Col.]. Bulletin de la Société Entomologique de France 1896: 352–355.

[B10] Pic M (1912a) Coleopterorum Catalogus. Pars 41 – Ptinidae. In: Junk W, Schenkling S (Eds) Coleopterorum Catalogus. W. Junk, Berlin, 46 pp.

[B11] Pic M (1912b) Coleopterorum Catalogus. Pars 48 – Anobiidae. In: Junk W, Schenkling S (Eds) Coleopterorum Catalogus. W. Junk, Berlin, 92 pp.

[B12] Pic M (1915) Descriptions abrégées diverses. Mélanges Exotico-entomologiques 12: 3–20.

[B13] Pic M (1926) Nouveautes diverses. Mélanges Exotico-entomologiques 45: 1–32.

[B14] Pic M (1934) Nouveautes diverses. Mélanges Exotico-entomologiques 63: 1–36.

[B15] Pic M (1936) Nouveaux Coléoptères de l’Afrique Occidentale. Revue de Zoologieet de Botanique Africaines 29: 34–36.

[B16] Pic M (1947) Coléoptères du globe (suite). L’Échange, Revue Linnéenne 63: 1–4.

[B17] Pic M (1949) Nouveaux Coléoptères de Madagascar. Mémoires de L’Institut Scientifique de Madagascar, Série A 3: 341–345.

[B18] Pic M (1952) Mission A. Villiers au Togo et au Dahomey (1950) VI. Coléoptères diverses. Bulletin de l’Institut Français d’Afrique Noire 14: 97–119.

[B19] Pic M (1953) Coléoptères nouveaux de Madagascar. Mémoires de l’Institut Scientifique de Madagascar, Série E 3: 253–278.

[B20] White RE (1974) Type-species for world genera of Anobiidae (Coleoptera). Transactions of the American Entomological Society 99: 415–475.

